# Integrative Transcriptomic and Evolutionary Analysis of Drought and Heat Stress Responses in *Solanum tuberosum* and *Solanum lycopersicum*

**DOI:** 10.3390/plants14243851

**Published:** 2025-12-17

**Authors:** Eugeniya I. Bondar, Ulyana S. Zubairova, Aleksandr V. Bobrovskikh, Alexey V. Doroshkov

**Affiliations:** 1 Institute of Fundamental Biology and Biotechnology, Siberian Federal University, 660041 Krasnoyarsk, Russia; 2 Federal Research Center “Krasnoyarsk Science Center of the Siberian Branch of the Russian Academy of Sciences”, 660036 Krasnoyarsk, Russia; 3 The Federal Research Center Institute of Cytology and Genetics, Siberian Branch of the Russian Academy of Sciences, 630090 Novosibirsk, Russia; ulyanochka@bionet.nsc.ru (U.S.Z.); avb@bionet.nsc.ru (A.V.B.); 4 Department of Information Technologies, Novosibirsk State University, 630090 Novosibirsk, Russia; 5 Department of Natural Sciences, Novosibirsk State University, 630090 Novosibirsk, Russia

**Keywords:** potato, tomato, gene regulatory networks, transcriptomic meta-analysis, orthologous genes, differential gene expression, transcription factors, co-expression analysis, stress adaptation, systems biology

## Abstract

Abiotic stresses such as drought and heat severely constrain the growth and productivity of Solanaceae crops, including potato (*Solanum tuberosum* L.) and tomato (*Solanum lycopersicum* L.), yet the conserved regulatory mechanisms underlying their stress adaptation remain incompletely understood. Here, we performed an integrative meta-analysis of publicly available transcriptomic datasets, complemented by comparative and evolutionary analyses across the *Solanum* genus. Functional annotation revealed coordinated transcriptional reprogramming characterized by induction of protective processes, including molecular chaperone activity, oxidative stress responses, and immune signaling, accompanied by repression of photosynthetic and primary metabolic pathways, reflecting energy reallocation under stress conditions. Promoter motif and transcription factor enrichment analyses implicated the bZIP, bHLH, DOF, and BBR/BPC families as central regulators of drought- and heat-induced transcriptional programs. Orthogroup inference and $K_{a}/K_{s}$ analysis across representative *Solanum* species demonstrated a predominance of purifying selection, indicating evolutionary conservation of regulatory network architecture. Integration of motif occurrence, co-expression profiles, and protein–protein interaction data enabled reconstruction of regulatory networks and identification of conserved hub transcription factors coordinating stress responses. Comparative analysis revealed distinct but conserved transcriptional signatures for heat and drought shared between potato and tomato, indicative of conserved abiotic stress strategies across Solanaceae.

## 1. Introduction

*Solanaceous crops*, particularly *Solanum tuberosum* (potato) and *Solanum lycopersicum* (tomato), represent cornerstone species in global agriculture, ranking among the ten most economically and nutritionally significant food crops. Beyond their economic relevance, these species are key models for dissecting the genetic and physiological mechanisms underlying plant responses to environmental constraints due to their well-characterized genomes and extensive agronomic data.

However, global climate change represents an escalating challenge to the stability of *Solanum* crop yields. Projections suggest that an increase in mean global temperatures by 1–7 °C by the end of the century will profoundly alter precipitation regimes and regional weather patterns [1,2]. In drought-prone regions, this shift is expected to exacerbate water scarcity and intensify abiotic stress, particularly in drought-sensitive crops such as potato [3,4]. These environmental perturbations are predicted to diminish tuber yield and quality, reducing dry matter accumulation while increasing the content of reducing sugars [5]. Concurrently, increased evapotranspiration and irregular rainfall will heighten crop water demand, collectively imposing prolonged and more frequent drought episodes that threaten agricultural productivity and food security.

Potato plants (*Solanum tuberosum*) are particularly susceptible to both drought and heat stresses [6,7], mainly due to its shallow, adventitious root system [8] with low water absorption ability, and adaptation to moderate temperatures [9,10,11]. The drought itself inhibits plant growth [12], shortens growing period [13], and reduces amount [14] and size [15] of the tubers. Heat stress that often accompanies water deficit also has critical influence on potato yield [16,17,18]. The optimum temperature for the initiation and bulking of the underground tubers are known to be 15–20 °C [11]. Chronic increase in temperature to 13.8–33 °C leads to decrease in ABA and auxin levels in leaves and tuber tissues, and causes reduction in the average number of tubers per plant [19], greatly affecting the productivity [20]. Heat stress delays tuber set and growth and causes tuber quality problems such as deformation and necrosis [7,21,22].

Tomato plants (*Solanum lycopersicum*) are highly susceptible to abiotic stresses such as high temperature and drought, which severely impair vegetative growth, reproductive development, and overall yield [23]. Heat stress negatively impacts tomato growth, limits nutrient availability, inhibits photosynthesis, impairs reproduction, denatures proteins, disrupts signaling pathways, damages cell membranes, and causes excessive production of reactive oxygen species [23]. All of these factors are toxic to tomato plants. High-temperature stress disrupts pollen viability, fruit set, and photosynthetic performance, leading to substantial yield losses [24,25]. One of the problems facing tomatoes under heat stress is oxidative stress and fruit set. Currently, this problem has been proposed to be addressed by using mutant parthenocarpic tomato varieties [26]. Under heat stress, the parthenocarpic genotypes studied exhibited lower electrolyte loss characteristics and lower $H_{2}O_{2}$ concentrations, as well as higher antioxidant activity compared to wild tomatoes under heat stress. Although thermotolerance is present among tomato varieties, the mechanisms underlying this tolerance require further study. The precise roles and interactions of signaling pathways in response to heat stress remain unclear and require systematic integration of diverse data [23]. The authors note that significant progress remains to be made in identifying mechanisms that can be modified to create plants that are resilient to current heat stress and resilient to the prospect of increased global warming. The known part of molecular basis of thermotolerance in tomato involves a hierarchical network of heat shock transcription factors (HSFs): HsfA1a acts as the master regulator initiating the early heat response, HsfA2 mediates acquired thermotolerance during prolonged exposure, while HsfB1 functions as both co-activator and repressor to fine-tune the balance between growth and protection [27,28]. Additional regulatory layers include C2H2-type zinc finger proteins and microRNAs that orchestrate transcriptional reprogramming under both individual and combined heat and drought stress [29,30]. Exogenous application of ascorbic acid and inoculation with heat-tolerant plant-growth-promoting rhizobacteria further mitigate stress damage by enhancing antioxidant defenses and stabilizing cellular homeostasis [31,32]. Together, these findings establish tomato as an important model for studying molecular and physiological adaptations to environmental stress.

Unlike visible phenotypic traits or metabolite accumulation that often can be explained by a few genetic variants, a plant’s response to abiotic stress is a complex, multilayered process largely governed by transcriptional reprogramming. Although extensive progress has been made in elucidating the molecular mechanisms of stress tolerance in model plants such as *Arabidopsis thaliana*, the specific features of stress responses in *Solanum* species remain insufficiently characterized. Recent genome-wide transcriptomic studies have identified numerous candidate genes involved in responses to heat stress and water deficit; however, most individual experiments provide limited mechanistic insight, are restricted to a narrow set of genotypes, and often focus on a single environmental condition. Consequently, the broader architecture of transcriptional regulation, including gene co-expression networks and major transcription factors with their downstream targets, remains poorly understood in potato and tomato. Addressing these gaps requires an integrative analytical framework capable of comparing heterogeneous transcriptomic datasets to reveal conserved regulatory patterns and clarify shared molecular pathways underlying stress adaptation. To date, a number of transcriptomic studies have investigated changes in gene expression profiles under heat and water deficit stress conditions in both *S. tuberosum* [33,34,35,36,37,38,39] and *S. lycopersicum* [27,40,41,42,43,44]. However, comparative analyses examining conserved and species-specific transcriptional responses in these crops remain limited [45]. A systematic meta-analysis of stress-induced transcriptomes can reveal conserved regulatory patterns and clarify shared molecular pathways underlying adaptation. Previously, we demonstrated the effectiveness of this bioinformatic strategy by integrating multiple transcriptomic datasets on high-light stress in *A. thaliana*, first across five independent experiments [46] and subsequently across twenty-one datasets encompassing 58 distinct light-stress conditions [47]. These analyses collectively identified 218,000 instances of differentially expressed genes corresponding to 19,000 unique loci, delineating distinct transcriptional programs between short- and long-term light stress responses and refining the understanding of core regulatory modules. Drawing on the demonstrated effectiveness of this approach in *A. thaliana*, we reasoned that this integrative framework may provide valuable insights into conserved and species-specific transcriptional programs underlying abiotic stress adaptation in *Solanum* species.

In today’s world of widespread access to diverse data, studies involving large-scale pan-omic analyses are essential for understanding the variability arising from complex interactions between genes, proteins, metabolites, and regulatory networks within species [48]. Comparative studies of the composition of essential, variable, and rare or unique components in different samples help identify new genes, proteins, and metabolites responsible for stress resistance. For example, in tomato, integrating omics data into a single, unified picture helped uncover mechanisms of resistance to biotic stress [49] and identify protein-coding genes and non-coding RNAs that could be targets for genetic editing to create pathogen-resistant varieties. More powerful methods include approaches that integrate omics data across a sample of related species [50,51]. Matthias Benoit and co-authors [52] conducted extensive work structuring pangenome data for the genus Solanum, integrating functional genomics and pangenetics. The authors demonstrated that at high levels of chromosomal synteny, gene duplication and subsequent paralogous diversification are the main obstacles to the predictability of genotype-phenotype relationships. For example, they found that the loss of the redundant paralog of the *CLAVATA3 (CLV3)* gene can be compensated for by tandem duplication followed by gene fusion, creating a single fused *CLV3* allele capable of regulating fetal organ number in conjunction with the enzymatic gene controlling the same trait [52]. Thus, when making interspecific comparisons, it is necessary to take into account both the composition of orthologs of each species and the characteristics of substitution accumulation.

In this study, we perform a meta-analysis of differential gene expression under drought and heat stress in *Solanum tuberosum* and *Solanum lycopersicum*, integrating data from several transcriptomic experiments. Furthermore, we analyze orthogroups among multiple *Solanum* species to identify conserved and divergent responses to abiotic stress, aiming to uncover core stress response modules and species-specific adaptations.

## 2. Results

### 2.1. Search and Selection of Relevant Transcriptomic Experiments for Analysis

To comprehensively investigate the transcriptomic responses of *Solanum tuberosum* and *Solanum lycopersicum* to drought and heat stress, we collected and processed all publicly available RNA-seq datasets for these species. Transcriptomic experiments were retrieved from the NCBI Sequence Read Archive (https://www.ncbi.nlm.nih.gov/sra, (accessed on 29 October 2025)) and BioProject (https://www.ncbi.nlm.nih.gov/bioproject/, (accessed on 29 October 2025)) databases using targeted searches with the keywords “drought,” “water deficit,” “dehydration,” “heat,” and “high temperature,” in combination with the species taxonomy identifiers txid4081 and txid4113. The resulting dataset list was manually curated to remove non-relevant, redundant, or low-quality experiments.

After the initial screening and quality control steps, a total of 450 RNA-seq samples were selected for downstream analysis. Specifically, the dataset included 48 transcriptomic samples corresponding to three independent experiments on heat stress in potato, 184 samples from six drought stress experiments in potato, 143 samples from six heat stress experiments in tomato, and 75 samples from six drought stress experiments in tomato (listed in Table 1). Detailed information on all analyzed experiments, including accession numbers, sample descriptions, and stress treatment conditions, is provided in Supplementary File S1.

**Figure 1:**
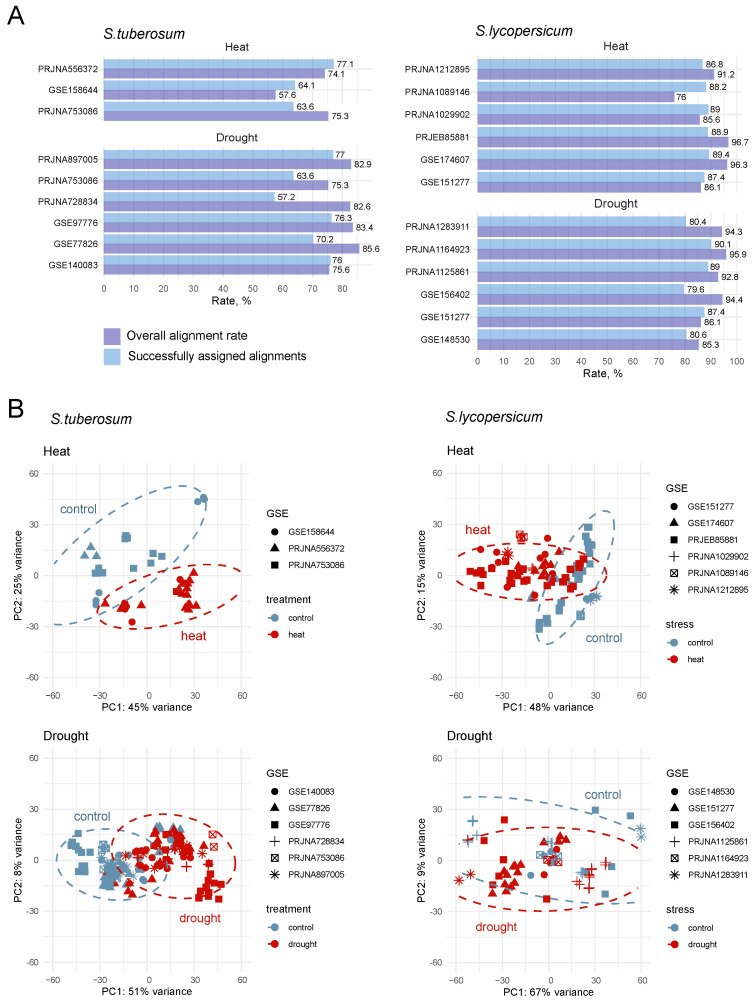
Summary of RNA-seq data quality and sample variance for *S. tuberosum* and *S. lycopersicum* under drought and heat stress. (**A**) Overall alignment rates obtained with Hisat2 (pale purple) and percentages of successfully assigned reads calculated with featureCounts (pale blue) across all analyzed experiments. (**B**) Principal component analysis (PCA) of normalized expression data after batch effect removal using the removeBatchEffect function from the limma package. The plot displays the first and second principal components (PC1 and PC2), with the proportion of explained variance indicated on each axis. Stress-treated samples are shown in red, and control samples in pale blue.

The overall summary of sequencing depth, mapping statistics, and sample variance for both species under drought and heat stress is presented in Figure 1. Across all samples, the average alignment rate obtained with Hisat2 was 92% for tomato and 77% for potato. The mean proportion of successfully assigned reads, as calculated by featureCounts, reached 85% for tomato and 70% for potato (Figure 1A; Supplementary Table S2). Principal component analysis (PCA) of normalized expression profiles, performed separately for each species and stress condition after batch-effect correction using the removeBatchEffect function from the limma package, revealed clear clustering of biological replicates within each dataset (Figure 1B).

### 2.2. Differential Gene Expression Analysis

Differential expression analysis of the combined RNA-seq datasets using DESeq2 identified a total of 4536 significant genes in *S. tuberosum* under heat stress and 5484 under drought stress. In *S. lycopersicum*, 5527 genes were found to be significantly differentially expressed under heat stress, whereas 440 DEGs were detected under drought stress (Supplementary Table S2).

**Figure 2:**
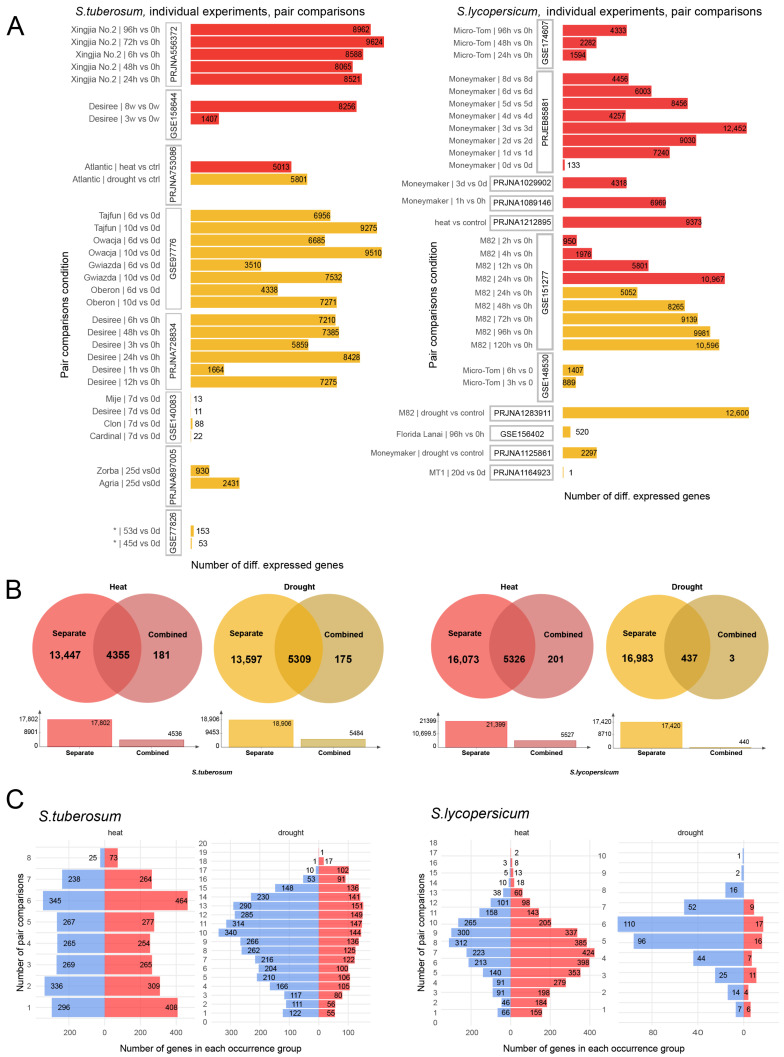
Differential gene expression analysis for *S. tuberosum* and *S. lycopersicum* under drought and heat stress. (**A**) Numbers of differentially expressed genes (DEGs) identified in each pairwise comparison from individual experiments. Experiments involving heat stress are shown in red, and those involving drought stress in yellow. The cultivar and exposure time are indicated on the Y-axis, and the total number of DEGs is shown at the right end of each bar. * Milva, Alegria, Desiree and Saturna cultivars were used for GSE77826 experiment. (**B**) Overlap between DEGs identified using two analytical approaches: individual-experiment analysis and combined-experiment analysis. Each Venn diagram represents one species–stress combination (*S. tuberosum* or *S. lycopersicum* under drought or heat stress). Bars below the Venn diagrams indicate the size of each DEG set (i.e., the number of genes). (**C**) Distribution of DEGs across occurrence groups. DEGs were obtained from the intersection of the two analytical approaches, and groups were defined by the number of pairwise comparisons in which a gene was detected as differentially expressed. Each group includes both upregulated (red) and downregulated (blue) genes, displayed separately in the plot. The number of pairwise comparisons is shown on the Y-axis, and the counts of up- and downregulated genes within each group are displayed at the end of the bars.

In parallel, differential expression was analyzed within each individual experiment to assess the reproducibility of transcriptional responses under comparable stress conditions (Supplementary Table S2; Figure 2A). Genes were ranked according to the frequency with which they were detected as DEGs across independent pairwise comparisons. This approach allowed us to identify groups of genes that were consistently differentially expressed across multiple experiments, reflecting stable components of the stress response network. Specifically, in *S. lycopersicum*, DEGs detected in at least 10 out of 17 pairwise comparisons under heat stress were retained. In *S. tuberosum*, DEGs identified in at least 6 out of 8 pairwise comparisons under heat stress and in at least 13 out of 18 comparisons under drought stress were selected.

The overlap between these consistently identified DEGs from individual experiments and the DEGs obtained from the combined-analysis approach is shown in Figure 2B. This intersection was considered a validated set of differentially expressed genes, representing high-confidence stress-responsive candidates. From this validated DEG set, the top 1000 genes (500 upregulated and 500 downregulated) were selected for each species–stress combination for further analysis. Within each occurrence group, genes were ordered by the absolute value of their $\log_{2}$ fold change (|LFC|), ensuring that the most strongly regulated genes were prioritized within each group. The distribution of DEGs across occurrence groups, separated into up- and downregulated subsets, is illustrated in Figure 2C.

For *S. lycopersicum* under drought stress, only 440 DEGs were identified in the combined analysis; therefore, all of them were included in downstream analyses. Overall, integrating the two analytical strategies (Figure 2) enabled us to identify a robust and reproducible core set of stress-responsive genes while minimizing experiment-specific variability.

### 2.3. Functional and Regulatory Analysis of Differentially Expressed Genes Under Drought and Heat Stress

#### 2.3.1. Functional Annotation of Differentially Expressed Genes

**Figure 3:**
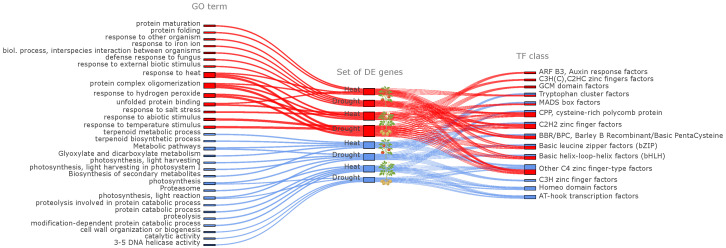
Sankey diagram illustrating the relationships between the top differentially expressed genes (DEGs) in each species–stress combination, their enriched Gene Ontology (GO) terms, and transcription factor (TF) classes predicted in the promoters of these DEGs. The central nodes represent eight DEG groups, defined by the combination of species (*S. tuberosum* or *S. lycopersicum*), stress type (drought or heat), and regulation direction (up- or downregulation, shown in red and blue, respectively). Links on the left connect each DEG group to significantly enriched GO terms, whereas links on the right connect DEG groups to associated TF classes.

Functional enrichment analysis was performed using the clusterProfiler package in R (v. 4.4.1) to identify overrepresented Gene Ontology (GO) terms among the top differentially expressed genes (DEGs) in each species–stress combination. The analysis revealed distinct but partially overlapping sets of biological processes associated with upregulated and downregulated genes (Figure 3).

Upregulated DEGs in both *S. tuberosum* and *S. lycopersicum* were predominantly enriched in GO terms related to cellular protection, protein quality control, and stress-responsive signaling pathways. These enriched categories can be grouped into three major functional modules:

(1) Protein quality control and protection against denaturation. Enriched terms included “protein folding,” “protein maturation,” “unfolded protein binding,” and “protein complex oligomerization.” The strong induction of chaperones (e.g., *HSP70*, *HSP90*) is a cornerstone of thermotolerance in crop plants. For *S. lycopersicum* (tomato), this response is vital for protecting the photosynthetic apparatus and reproductive development during heat waves, which are major causes of fruit set failure and yield loss [26,53]. In *S. tuberosum* (potato), a crop highly sensitive to soil temperature fluctuations, robust protein quality control is essential for protecting the enzymatic pathways involved in starch biosynthesis and tuberization, processes that are critically disrupted by heat stress.

(2) Protection against oxidative damage and activation of stress-associated pathways. Key enriched terms included “response to hydrogen peroxide,” “response to iron ion,” “defense response to fungus,” and “response to external biotic stimulus.” This indicates a coordinated activation of antioxidant and immune defense mechanisms. The cross-talk between abiotic and biotic stress responses, as seen in the enrichment of defense-related terms, is particularly relevant for these species. In tomato, drought and heat stress can predispose plants to fungal pathogens like *Botrytis cinerea* [54,55,56]; the concurrent activation of these pathways may represent a pre-emptive defense priming. Furthermore, managing ROS signaling is crucial for fruit development in tomatoes [23,57], highlighting the central role of these antioxidant systems in agronomically important traits.

(3) Stress-specific adaptive responses. Processes such as “response to heat,” “response to temperature stimulus,” “response to salt stress,” and “response to abiotic stimulus” confirm that both species deploy broad-spectrum stress tolerance mechanisms. The co-enrichment of “response to salt stress” is noteworthy, as it suggests the activation of shared osmotic adjustment pathways, which could be leveraged to improve tolerance to multiple abiotic constraints in these species through breeding.

Conversely, downregulated DEGs were enriched in categories related to primary metabolism, photosynthesis, and growth, reflecting an adaptive metabolic shift towards energy conservation under adverse conditions. Together, these results indicate that Solanaceae plants under heat and drought stress activate a coordinated transcriptional program that strengthens proteostasis, enhances redox balance, and suppresses growth-related processes in favor of survival.

#### 2.3.2. Identification of Regulatory Motifs and Transcription Factor Classes

To elucidate potential upstream regulatory mechanisms controlling the expression of stress-responsive genes, promoter regions of the top 1000 DEGs (500 up- and 500 downregulated genes per species–stress combination) were extracted using bedtools [58]. *De novo* motif discovery was performed with the XSTREME tool from the MEME Suite, followed by functional annotation of motifs using TOMTOM. This approach enabled the identification of sequence motifs corresponding to known transcription factor (TF) binding sites and their classification according to TF families.

After statistical filtering based on the criterion *q*-value <0.05, 305 significant motif–set associations were retained, including 119 unique motifs and 281 unique motif–set combinations. The annotated motifs were classified into 14 TF families, many of which are known regulators of stress, photomorphogenesis, and metabolic adaptation. Among the most represented families were basic helix–loop–helix (bHLH) and basic leucine zipper (bZIP) factors, consistent with their well-established roles in abiotic and biotic stress responses (Figure 3).

Several motif–TF associations were particularly notable: the *BPC6*-like motif (BBR/BPC family) was enriched in the heat-associated set of downregulated genes in *S. lycopersicum*, suggesting repression of heat-response genes; the *DOF3.4*-like motif (DOF family) appeared in the drought-associated set of upregulated genes in *S. lycopersicum*, reflecting its known involvement in drought response; a G-box–like motif (CACGTG), characteristic of bZIP, bHLH, and GBF factors, was found in the drought-associated set of downregulated group of genes; and A/T-rich motifs (e.g., TTAAAAA, TATTTTAAAT) were prevalent in the heat-associated sets of up- and downregulated genes in *S. tuberosum*, possibly indicating bZIP/ERF-like binding preferences or promoter composition bias.

Overall, the comparative analysis of promoter motifs and TF class distribution highlights a complex and multilayered regulatory landscape underlying transcriptional stress responses in Solanaceae. The Sankey diagram (Figure 3) integrates the relationships between DEG groups, enriched GO terms, and transcription factor classes, providing a unified overview of functional and regulatory modules involved in plant adaptation to drought and heat stress.

### 2.4. Comparative Genomic and Orthogroup Analysis of Solanaceae Species

#### 2.4.1. Identification of Orthologous Groups

To place transcriptomic results in an evolutionary context and assess the conservation of stress-responsive genes across related taxa, a comparative genomic analysis was conducted using representative Solanaceae species and several wild potato genomes. The analysis aimed to identify orthologous gene groups, detect lineage-specific gene expansions, and characterize selective constraints influencing the evolution of stress-associated genes in *S. tuberosum* and *S. lycopersicum*.

Phylogenetic orthology was inferred using OrthoFinder [59] based on predicted proteomes from 11 plant genomes. The dataset included eight reference Solanaceae genomes retrieved from the PLAZA database (https://bioinformatics.psb.ugent.be/plaza.dev) and three wild potato genomes (*Solanum neorossii* PG6243, *S. pinnatisectum* PG1013, and *S. burkartii* PG4005) reported in [60]. Protein sequences were extracted from genome FASTA and GFF annotation files, filtered to remove incomplete or truncated transcripts, and merged into nonredundant loci. Orthogroups were computed for both the full set of *Solanum* species and a representative subset of model Solanaceae genomes (*Nicotiana tabacum*, *Petunia axillaris*, *Capsicum annuum*, *S. lycopersicum*, *S. pennellii*, and *S. tuberosum*). The complete list of analyzed species and genome sources is provided in Supplementary File S3.

**Figure 4:**
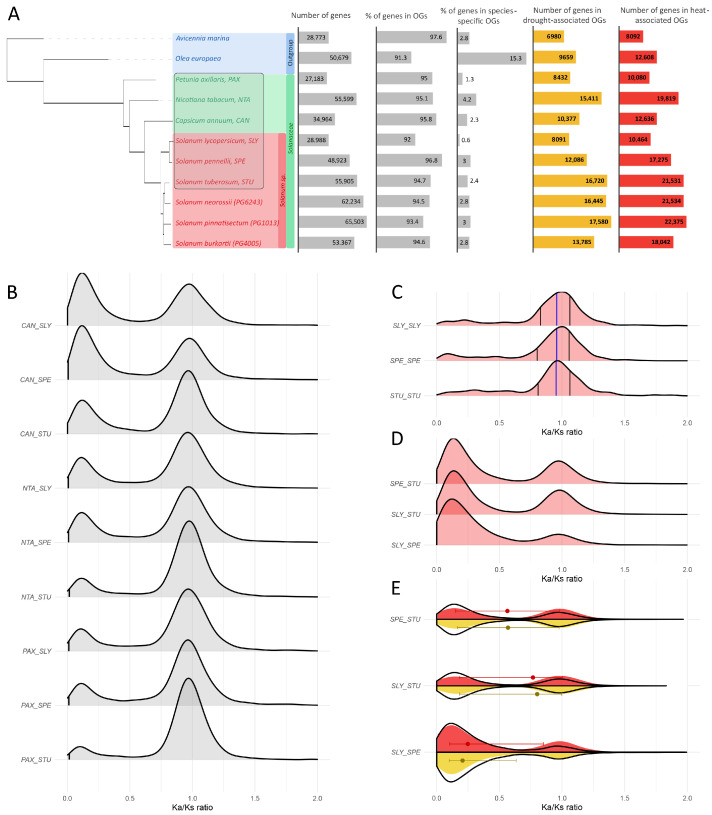
Phylogenetic relationships, orthogroup statistics, and $K_{a}/K_{s}$ ratio distributions for selected plant genomes. (**A**) Phylogenetic relationships and orthogroup statistics for 11 plant genomes analyzed using OrthoFinder. Leaves are highlighted with colored rectangles indicating taxonomic groups (red, Solanum spp.; green, Solanaceae; blue, outgroup). On the right, five horizontal bar charts summarize gene content and orthogroup information for each species: (1) total number of genes, (2) percentage of genes assigned to orthogroups, (3) percentage of genes in species-specific orthogroups, (4) number of genes in drought-associated orthogroups, and (5) number of genes in heat-associated orthogroups. Each bar chart includes 11 bars corresponding to the species in the phylogenetic tree, aligned horizontally for direct comparison. (**B**) $K_{a}/K_{s}$ ratio distributions for six species highlighted in panel A. (**C**) $K_{a}/K_{s}$ ratio distributions for paralogous genes in *S. lycopersicum*, *S. pennellii*, and *S. tuberosum*. (**D**) $K_{a}/K_{s}$ ratio distributions for orthologous genes in *S. lycopersicum*, *S. pennellii*, and *S. tuberosum*. (**E**) Split violin plot showing $K_{a}/K_{s}$ ratios for orthologous genes based on DEGs derived from stress-associated HOGs. The red upper half of each split violin represents heat-associated genes, and the yellow lower half represents drought-associated genes. Solid black lines in each half indicate the $K_{a}/K_{s}$ distributions for genes not belonging to stress-associated HOGs.

The resulting orthogroup statistics and phylogenetic relationships among the 11 analyzed genomes are summarized in Figure 4, and detailed orthogroup composition data are presented in Supplementary File S4. Across all species, more than 90% of protein-coding genes were assigned to orthogroups, confirming the robustness of clustering. The total number of predicted genes varied substantially among species, from 27,183 in *P. axillaris* to 65,503 in *S. pinnatisectum*. A generally higher gene count was characteristic of *Solanum* species, as well as *N. tabacum* and *Olea europaea*, consistent with previously reported whole-genome and segmental duplications within these evolutionary lineages.

Among *Solanum* species, both cultivated and wild potatoes exhibited higher total gene counts compared with other Solanaceae members. In contrast, *S. lycopersicum* contained approximately half as many genes as *S. tuberosum* (28,988 vs. 55,905). Accordingly, the proportion of species-specific orthogroups in *S. lycopersicum* was markedly lower (0.6%) than in other *Solanum* species (2.8–3.0%). These unique orthogroups likely originated through genome rearrangements following duplication events, activation of previously non-coding regions, or gene fusion and fission processes.

This pattern suggests that homology-based inference alone may underestimate species-specific adaptations within stress-response networks, underscoring the importance of direct transcriptomic analysis in *S. tuberosum*. To further examine the representation of stress-associated genes, orthogroups containing at least one stress-related gene in any species were identified, and their distribution across taxa was analyzed (Figure 4A). The number of genes in stress-associated orthogroups correlated with total gene counts across species (Pearson’s linear correlation, p<0.005). Approximately one-third of all genes belonged to orthogroups that included genes differentially expressed under drought conditions, with a similar trend observed for heat stress.

#### 2.4.2. Evolutionary Dynamics and Selective Constraints in Orthologous Genes

To explore evolutionary patterns within the Solanaceae family, we compared nonsynonymous-to-synonymous substitution rate ratios ($K_{a}/K_{s}$) among orthologous and paralogous gene pairs across six representative species: *Capsicum annuum*, *Nicotiana tabacum*, *Petunia axillaris*, *Solanum lycopersicum*, *S. pennellii*, and *S. tuberosum*. Only hierarchical orthogroups containing genes from all species were retained to ensure comparable evolutionary depth. The full list of orthogroup assignments and $K_{a}/K_{s}$ estimates is provided in Supplementary Files S4 and S5.

The $K_{a}/K_{s}$ distributions obtained by comparing species of the genus *Solanum* (*S. lycopersicum*, SLY; *S. pennellii*, SPE; *S. tuberosum*, STU) with more distantly related Solanaceae taxa (*C. annuum*, CAN; *N. tabacum*, NTA; *P. axillaris*, PAX) are shown in Figure 4B. Across these comparisons, the genes displayed a broad range of substitution rate ratios from values close to zero to slightly above 1.0, indicating the coexistence of strong purifying and relaxed selection regimes. However, no excess of genes with elevated nonsynonymous substitution rates ($K_{a}/K_{s}\gg 1$) was observed, suggesting the absence of widespread positive selection and supporting the predominance of conservative evolutionary dynamics across the family.

Most orthologous gene pairs showed $K_{a}/K_{s}$ values centered around 1.0, consistent with neutral evolution, whereas a smaller subset fell within the 0.1–0.3 range, corresponding to negative selection. Within-species paralog comparisons in *S. lycopersicum* (SLY), *S. pennellii* (SPE), and *S. tuberosum* (STU) yielded unimodal distributions centered near 1.0, indicative of neutral divergence following gene duplication (Figure 4C). The distributions did not differ significantly among the three species (Wilcoxon rank-sum test, p>0.05).

Pairwise comparisons between the three *Solanum* species (SLY vs SPE, STU vs SLY, SPE vs STU) revealed a distinct bimodal $K_{a}/K_{s}$ distribution (Figure 4D), significantly different from all previous datasets (Wilcoxon test, p<0.05). The first peak (0.1–0.3) corresponded to strong purifying selection, while the second peak near 1.0 reflected neutral evolution. This pattern suggests the coexistence of conserved loci essential for fundamental cellular functions and more variable genes that may contribute to adaptive diversification. Among these, the most pronounced conservation was observed for the *S. lycopersicum*–*S. pennellii* pair, which represents the shortest evolutionary distance (p<0.05 compared to other species pairs).

Stress-associated orthogroups exhibited distributions similar between drought and heat stress (Figure 4E), but when compared to all other genes, showed a moderate shift toward higher $K_{a}/K_{s}$ values in the SLY–STU pair (Wilcoxon test, p<0.05). Although purifying selection remained the dominant trend, this upward shift indicates a slight relaxation of evolutionary constraints and possibly faster sequence evolution in stress-responsive genes.

In comparisons among more distant Solanaceae taxa, the entire $K_{a}/K_{s}$ distribution shifted toward higher values (Figure 4B), reflecting the gradual weakening of selective pressure with increasing phylogenetic distance. Nevertheless, all *Solanum*-specific comparisons consistently showed dominant low $K_{a}/K_{s}$ values, confirming that purifying selection remains the prevailing evolutionary mode within this genus.

Overall, these results demonstrate that most genes in Solanaceae evolve under purifying selection, which persists across evolutionary distances of up to approximately 7 million years, consistent with the divergence time between *S. lycopersicum* and *S. tuberosum* (TimeTree median 8.0 MYA; adjusted 7.1 MYA) [61]. Paralogous genes evolve primarily under neutral drift, whereas stress-associated genes show mild relaxation of selective pressure. This long-term evolutionary conservatism of core stress-response components provides a robust framework for cross-species inference of regulatory mechanisms and supports the stability of transcriptional and signaling pathways underlying environmental adaptation.

### 2.5. Gene Network Analysis of Stress Response

**Figure 5:**
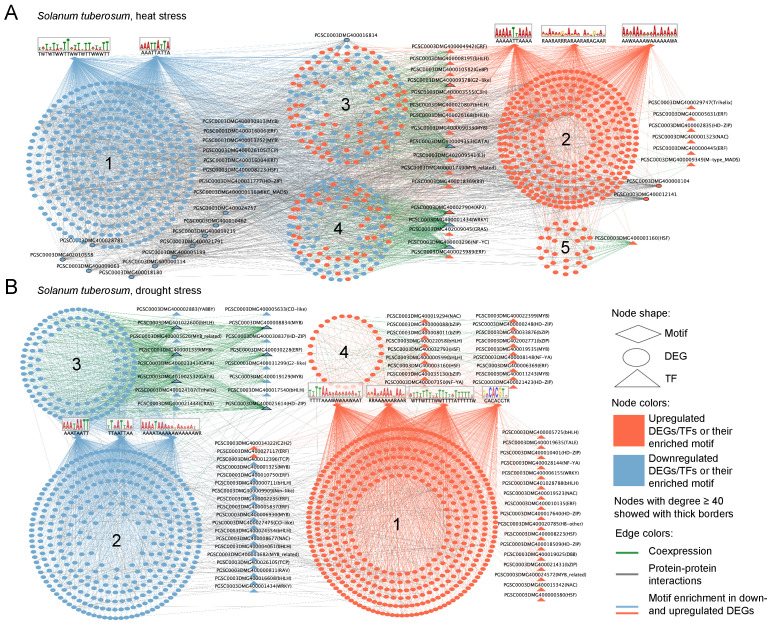
Integrated GRNs reconstructed for *Solanum tuberosum* under (**A**) heat and (**B**) drought stress conditions. Nodes are colored according to expression pattern: red for upregulated and blue for downregulated genes. Node shapes indicate element type: circles represent differentially expressed genes (DEGs), triangles denote transcription factors (TFs), and rhombi correspond to TF binding site motifs. Edge colors reflect the source of evidence: green for co-expression, gray for protein–protein interactions (PPIs), and red or blue for motif enrichment in up- or downregulated DEGs, respectively.

**Figure 6:**
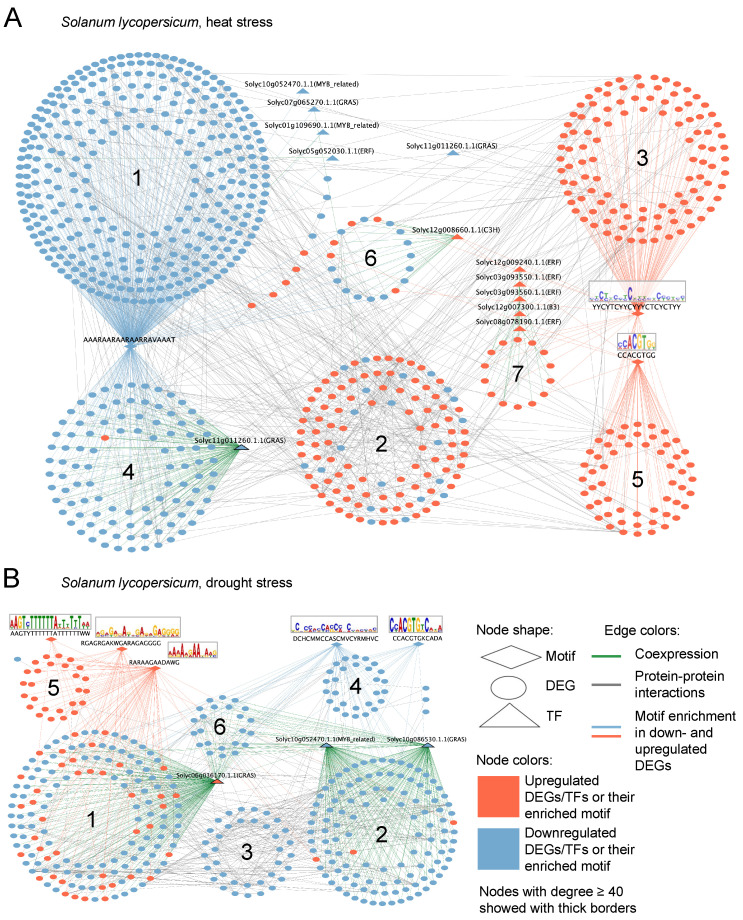
Integrated GRNs reconstructed for *Solanum lycopersicum* under (**A**) heat and (**B**) drought stress conditions. Nodes are colored by expression pattern: red for upregulated and blue for downregulated genes. Node shapes denote element type: circles for differentially expressed genes (DEGs), triangles for transcription factors (TFs), and rhombi for TF binding site motifs. Edge colors represent evidence type: green for co-expression, gray for protein–protein interactions (PPIs), and red or blue for motif enrichment in up- or downregulated DEGs, respectively.

Integration of motif-based, co-expression, and protein–protein interaction (PPI) data resulted in four comprehensive gene regulatory networks (GRNs) representing *S. tuberosum* and *S. lycopersicum* responses to drought and heat stress (Figure 5 and Figure 6). Functional annotations for all clusters in the reconstructed GRNs are provided in Supplementary Table S6. Below, we describe the largest clusters (containing 100 or more genes).

#### 2.5.1. *Solanum tuberosum*

The reconstructed GRN of *S. tuberosum* under heat stress (Figure 5A) comprised 961 genes and 3856 interactions, including 587 co-expression links, 1664 motif-based regulatory associations, and 1605 protein–protein interactions. Four major clusters were identified; their functional annotations are summarized in Supplementary Table S6.1.

The first cluster consists of 336 genes that were predominantly downregulated in response to heat. This cluster is enriched in processes related to catabolism (GO:0009056), proteolysis involved in protein catabolism (GO:0051603), and intracellular transport (GO:0046907). Two motifs were significantly enriched in the promoter regions of genes in this cluster: AAATTATTA and TWTWTWWTTWWTWTTWWWTT.

The second cluster includes 301 genes and six transcription factors that were coordinately upregulated under heat stress: *PGSC0003DMG400000445* (AP2/ERF), *PGSC0003DMG400009349* (MADS-box protein), *PGSC0003DMG400002835* (Homeobox protein), *PGSC0003DMG400005631* (AP2/ERF), *PGSC0003DMG400029747* (Trihelix), and *PGSC0003DMG400001323* (NAC). Three motifs were significantly enriched in the promoter regions of genes in this cluster: AAAAATTAAAA, RAARARRRARAARARAGAAR, and RAARARRRARAARARAGAAR. Functionally, this cluster is associated with the *response to heat* (GO:0009408), *response to oxidative stress* (GO:0006979), *macromolecule biosynthetic process* (GO:0009059), and *RNA metabolic process* (GO:0016070).

The third cluster is composed mainly of co-expression interactions linking 12 transcription factors to 140 target genes. It is enriched in the *ubiquitin-dependent protein catabolic process* (GO:0006511) and *vesicle-mediated transport* (GO:0016192). Remarkably, only one transcription factor, *PGSC0003DMG400009353* (GATA), was downregulated; this gene functions as the central hub of the cluster with 76 edges. The remaining transcription factors were upregulated, including three bHLH, two B3, and several others from different families. Overall, the cluster exhibits mixed regulation, with 67 downregulated and 73 upregulated genes.

The fourth cluster comprises five transcription factors and 124 associated genes, connected mainly through co-expression links. This cluster also displays mixed regulation (78 downregulated, 46 upregulated genes) and is functionally linked to transport processes (GO:0006810) and proteolysis (GO:0006508). Three transcription factors show high connectivity (greater than 40 edges): *PGSC0003DMG400027904* (AP2), *PGSC0003DMG400001434* (WRKY), and *PGSC0003DMG400003296* (NF-YC).

The reconstructed GRN of *S. tuberosum* under drought stress (Figure 5B) comprised 993 genes and 3498 interactions, including 450 co-expression links, 2668 motif-associated connections, and 380 protein-protein interactions. Six major clusters were identified, and their functional annotations are provided in Supplementary Table S6.2.

The first cluster is upregulated and includes 430 genes together with 17 transcription factors from diverse families. Most interactions in this cluster are motif-enriched, with four significantly overrepresented promoter motifs: GACACGTR, RRAAAAAARAAR, TTTTAAAWAWAAWAAT, and WTTWTTTWWTTTTATTTTTW. Functionally, the cluster is associated with protein metabolism (GO:0019538), response to stress (GO:0006950), and transport processes (GO:0006810).

The second cluster is downregulated and contains 340 genes and 17 transcription factors. Three motifs were significantly enriched in the promoter regions of genes in this cluster: AAATAATT, TTAATTAA, and AAAATAAAAAWAAAAAWR. This cluster is associated with *protein modification* (GO:0036211), *signal transduction and cell communication* (GO:0007165, GO:0007154), *response to hormone* (GO:0009725), and *response to light stimulus* (GO:0009416).

The third cluster, defined primarily by co-expression interactions, is also downregulated and includes 124 genes and 16 transcription factors, six of which act as central hubs (greater than 40 edges): *PGSC0003DMG401002532* (GATA), *PGSC0003DMG400025614* (HD-ZIP), *PGSC0003DMG401022600* (bHLH), *PGSC0003DMG400008834* (MYB), *PGSC0003DMG400030228* (ERF), and *PGSC0003DMG400001339* (MYB). This cluster is functionally enriched for protein phosphorylation (GO:0006468), developmental processes (GO:0032502), and cell wall organization or biogenesis (GO:0071554).

The enriched biological processes in heat GRN reflect well-known heat stress response: protein catabolism and proteolysis suggesting degradation of damaged proteins, oxidative stress response and RNA metabolism indicating cellular stress adaptation, ubiquitin-dependent pathways highlighting the need of proteins quality control, and transport processes which could facilitate metabolite and signal movement [62,63]. Identified TFs families such as AP2/ERF, NAC, WRKY, GATA, bHLH, MYB) are well-known in plant abiotic stress signaling. For instance, AP2/ERF and NAC families regulate stress-responsive gene expression widely under heat and drought, while WRKY factors often modulate oxidative stress and defense pathways [62,63,64]. The reconstructed GRN under drought stress captures the integration of drought signaling, hormonal regulation, structural and metabolic adaptation consistent with established molecular drought response in potato [65,66,67]. Under drought stress, involvement of TFs from such families as HD-ZIP, ERF, MYB, GATA indicate complex transcriptional networks controlling drought tolerance, hormone responses, protein metabolism, phosphorylation, and cell wall remodeling. These processes align with known drought adaptation mechanisms in potato and other plants [62]. Overall, the reconstructed GRNs reveal coordinated transcriptional programs integrating metabolic, hormonal, and developmental shifts that underpin potato’s drought and heat acclimation mechanisms.

#### 2.5.2. *Solanum lycopersicum*

The reconstructed GRN of *S. lycopersicum* under **heat stress** (Figure 6A) comprised 764 genes and 1335 interactions, including 149 co-expression edges, 625 motif-associated connections, and 561 protein–protein interactions.

The first cluster consists of downregulated DEGs and includes 335 genes and four transcription factors. Genes in this cluster share significant enrichment of a promoter motif AAARAARAARAARRAVAAAT. Functionally, this cluster is enriched in metabolic processes such as *small molecule metabolism* (GO:0044281), *carbohydrate metabolism* (GO:0005975), *lipid metabolism* (GO:0006629), and *terpenoid metabolism* (GO:0016114).

The second cluster contains 111 genes linked predominantly by protein–protein interactions according to STRING data. No specific transcription factors or regulatory motifs were identified for this cluster. It exhibits mixed regulation, with a predominance of upregulated genes (90 out of 111). Functionally, several genes in this cluster are associated with *biosynthetic processes* (GO:0009058), *gene expression* (GO:0010467), and *protein folding* (GO:0006457).

The third cluster, comprising 111 genes, is primarily upregulated. Genes in this cluster share a significantly enriched promoter motif YYCYTCYYCYYYCTCYCTYY. Seven genes within this cluster are associated with *RNA modification* (GO:0009451).

The fourth cluster, similar to the first one, is downregulated and linked to the same regulatory motif AAARAARAARAARRAVAAAT. However, it shows pronounced co-expression with the transcription factor *Solyc11g011260.1.1* (GRAS family). Genes in this cluster are enriched for *small molecule metabolism* (GO:0044281), *cellular response to chemical stimulus* (GO:0070887), and *cellular homeostasis* (GO:0019725).

The reconstructed network of *S. lycopersicum* under **drought stress** (Figure 6B) comprised 387 genes and 891 interactions, including 420 co-expression, 165 motif-enriched, and 306 protein–protein associations.

The first cluster exhibits mixed regulation and contains 151 genes co-expressed with the transcription factor *Solyc06g036170.1.1* (GRAS). The majority of genes in this cluster are downregulated (110 DEGs). Functional enrichment indicates involvement in metabolic processes including *small molecule metabolism* (GO:0044281), *carbohydrate metabolism* (GO:0005975), and *amino acid metabolism* (GO:0006520).

The second cluster is downregulated and comprises 116 genes co-expressed with two transcription factors: *Solyc10g052470.1.1* (MYB-related) and *Solyc10g086530.1.1* (GRAS). This cluster is enriched for genes associated with *phosphorus metabolism* (GO:0006793) and *photosynthesis* (GO:0015979).

The reconstructed GRN under heat stress highlights the suppression of metabolic pathways (carbohydrate, lipid, terpenoid, and small molecule). Additionally, downregulation linked with cellular response to chemical stimuli and homeostasis, suggesting a shift in basal metabolism and stress adaptation which coexpressed with a GRAS family TF. Upregulated clusters include genes related to biosynthesis, gene expression, protein folding, and RNA modification, reflecting active stress mitigation and repair responses under heat stress. This regulatory landscape fits with the known patterns of gene expression in tomato during heat stress [68,69,70]. Under drought stress, tomato shows a predominantly downregulated GRN with clusters coexpressed with TFs from GRAS and MYB-related families which linked to metabolism (carbohydrate, amino acid, phosphorus, small molecule) and photosynthesis. Overall, the drought GRN reflects tomato’s adaptive transcriptional suppression of growth and metabolic processes mediated by TFs to preserve water and ensure survival [71,72]. In summary, tomato’s heat stress response GRN demonstrates a balance of downregulated metabolic functions with activated protective and repair pathways, while the drought response relies on downregulation of metabolism and photosynthesis through MYB and GRAS TFs to optimize water usage and to enhance the stress endurance.

#### 2.5.3. Orthologs in Reconstructed Gene Networks

Taken together, the integrated network analysis reveals both shared molecular mechanisms underlying drought and heat responses and distinct evolutionary strategies of *S. tuberosum* and *S. lycopersicum*. The modular and hierarchical structure of these networks provides a systems-level perspective on stress adaptation and points to potential regulatory hubs for future functional studies.

A comparative analysis of the heat-responsive gene network components (961 genes in *S. tuberosum* and 764 genes in *S. lycopersicum*) identified 72 orthologous genes (see Supplementary Table S7), of which only three exhibited opposite regulation between the two species under heat stress. Among these orthologs, 41 genes were upregulated, representing several major functional groups.

- **Heat shock proteins:** three heat shock cognate 70 kDa proteins, two heat shock protein 83, and two additional heat shock proteins, which likely contribute to protein folding and protection under elevated temperature.
- **Transcriptional and RNA metabolism regulators:** two DNA-directed RNA polymerases, a serine/arginine-rich splicing factor, a DEAD-box ATP-dependent RNA helicase, a peptidyl–prolyl isomerase, and an AAR2 protein family member, indicating active post-transcriptional control of gene expression during heat stress.
- **Signaling components:** PERK1 kinase, WRKY transcription factor C, ethylene response factor ERF4, and a calmodulin-binding protein, reflecting the integration of hormonal and calcium-dependent signaling cascades in heat adaptation.

In contrast, 28 orthologous genes were downregulated, encompassing several categories of metabolic and organelle-associated functions.

- **Protein metabolism genes:** ubiquitin carrier protein, RING zinc finger protein, cysteine protease, protein phosphatase, heat shock protein DnaJ, protein translocase, and transitional endoplasmic reticulum ATPase, suggesting a reduction in protein turnover and degradation.
- **Other metabolic processes:** S-adenosyl-methionine-sterol-C-methyltransferase, delta-8 sphingolipid desaturase, fructose-1,6-bisphosphatase, glycerate dehydrogenase, amino acid transporter, 2-deoxyglucose-6-phosphate phosphatase, and starch synthase VI, which may indicate a shift in primary metabolism toward energy conservation.
- **Organelle-associated proteins:** chloroplast ferredoxin I, protein of the chloroplast import apparatus 2, DNA-directed RNA polymerase 2B, and mitochondrial small heat shock protein, pointing to altered plastid and mitochondrial functions under prolonged heat exposure.

Similarly, the comparison of drought-responsive GRNs (993 genes in *S. tuberosum* and 387 genes in *S. lycopersicum*) identified 33 orthologous genes, with only one showing opposite regulation. The majority of these orthologous DEGs (29) were downregulated, encompassing several key functional groups:

- **Photosynthesis- and chloroplast-related genes:** two chlorophyll a/b binding proteins, thylakoid soluble phosphoprotein, tetrapyrrole-binding protein, chloroplast fructose-1,6-bisphosphatase I, fructose-bisphosphate aldolase, PTAC16, and nitrite reductase, reflecting the suppression of photosynthetic activity under drought stress.
- **Cell wall and extracellular matrix metabolism:** arabinogalactan peptide 14, methionine-rich arabinogalactan, pectate lyase, and glycosyltransferase, suggesting the remodeling of the cell wall to prevent water loss.
- **Signaling and regulatory components:** serine/threonine protein kinase, P21-rho-binding domain-containing protein, extracellular calcium-sensing receptor, and ubiquitin-protein ligase BRE1, which indicate modulation of intracellular signaling and stress perception.
- **Amino acid and energy metabolism:** aspartate kinase, proline-rich protein, and ATP-binding protein, reflecting adaptive shifts in nitrogen and energy metabolism.
- **Lipid and membrane metabolism:** delta-9 desaturase, desaturase, and plastidial delta-12 oleate desaturase, likely associated with maintaining membrane fluidity under dehydration.
- **Antioxidant-related genes:** two germins and dihydrolipoyl dehydrogenase, implying reduced reactive oxygen species (ROS) detoxification capacity during drought.

Only three orthologous genes were upregulated under drought stress: *Triacylglycerol lipase*, *ATP-binding protein*, and *Ent-kaurene oxidase 2*, which may participate in lipid remodeling and hormonal signaling (gibberellin biosynthesis) in response to water deficit.

Overall, the presence of a shared core of stress-responsive orthologs between the two species indicates a conserved molecular framework for abiotic stress adaptation, while species-specific differences in regulatory patterns and pathway engagement reflect divergent evolutionary strategies shaped by their physiological and ecological contexts.

For instance, in heat stress responses, the expression patterns of heat shock proteins and antioxidant enzymes differ between tomato cultivars and desert species, like *Rhazya stricta* [73]. A recent comparison of the transcriptome response of *Solanum lycopersicum* and its wild relative, *Solanum pennellii* revealed that approximately 43% of orthologous genes displayed species-specific expression patterns in response to drought [74]. Even among different *Solanum tuberosum* cultivars, a total of approximately 7000 species-specific DEGs were identified in response to cold stress, indicating that cultivar-specific regulatory networks differ substantially [75]. Therefore, it is not surprising that we found approximately 10% overlap of core gene networks to be orthologous, based on the high evidence of high species specificity of transcriptomic response and the heterogeneous data available for such analysis.

Thus, the comparative analysis allowed us to describe the genetic basis for the observed general trends in resource redistribution from growth and active metabolism to maintaining plant viability. Multiple pairs of orthologous genes were identified that unidirectionally change their expression in response to the same stress factor. Under heat stress, we observe massive activation of proteostasis components (such as HSPs and others), post-transcriptional control, and stress signaling for the synthesis of protective macromolecules. Simultaneously, components associated with protein metabolism and organelle activity are suppressed, confirming the principle of ”economy and protection.” Under drought, components associated with photosynthesis, cell wall metabolism, and some antioxidant defenses are massively suppressed, while signaling and lipid metabolism are modulated to adapt to dehydration. The virtually complete absence of opposing regulation of orthologous genes, which are among the top 1000 differentially expressed genes in each species, emphasizes the universality of this basic strategy. Beyond this conserved framework, species-specific features were discovered: potato modulates intracellular communication and transport to a greater extent, while tomato more actively restructures primary metabolism. Regarding differential responses depending on the type of stress, a stronger activation of the oxidative response in potato can be noted in heat.

## 3. Discussion

Understanding orthologous gene relationships across *Solanum* species is essential for translating genomic and transcriptomic knowledge from well-studied model crops to their lesser-known relatives. Orthogroup analysis provides a robust framework to identify conserved gene families and evolutionary patterns that shape abiotic stress responses [76]. Recent comparative genome-wide studies across the genus have revealed both conserved and lineage-specific gene families involved in stress signaling, metabolism, and development [60,77,78,79]. These findings collectively lay the groundwork for integrating functional genomics with evolutionary biology to dissect how gene networks evolve under selective pressures imposed by the environment. Currently, extensive omics resources are available not only for cultivated potato and tomato (*S. tuberosum* and *S. lycopersicum*), but also for several of their wild relatives (*S. pinnatisectum*, *S. chacoense*, *S. jamesii*, *S. penelli*, and others) [60,74], which serve as valuable reservoirs of alleles conferring abiotic stress tolerance and highlight both the evolutionary and applied potential of comparative studies across the genus.

Within this broader context, our reconstructed GRNs illuminate both shared and species-specific aspects of abiotic stress response. The cross-species comparison highlights a dual pattern: on the one hand, the specificity of stress-induced transcriptional regulation within each species, and on the other, a remarkable degree of conservation among a subset of orthologous genes. Out of 105 orthologous genes identified across the networks, 101 exhibited the same direction of regulation under abiotic stress conditions. Such high concordance points to the presence of a conserved regulatory core that may function as an ancestral scaffold for stress adaptation. Notably, this core includes several transcription factors with well-established roles in plant stress signaling, including *WRKY transcription factor C* (*PGSC0003DMG400001434*/*Solyc02g088345*), *Ethylene response factor ERF4* (*PGSC0003DMG400016004*/*Solyc05g052030*), and a *DNA-binding protein* (*PGSC0003DMG402009541*/*Solyc08g006240*).

At the same time, network topology and hub composition reveal important species-specific differences. In *S. tuberosum*, the coexpression network under heat stress contained multiple hub transcription factors (*PGSC0003DMG400001434* (WRKY), *PGSC0003DMG400027904* (AP2), *PGSC0003DMG400009378* (G2-like), *PGSC0003DMG400003296* (NF-YC), and *PGSC0003DMG400009353* (GATA)), suggesting complex regulatory cross-talk between transcriptional modules. Under drought stress, the central nodes shifted toward other TF families, including *PGSC0003DMG400030228* (ERF), *PGSC0003DMG401002532* (GATA), *PGSC0003DMG400025614* (HD-ZIP), *PGSC0003DMG401022600* (bHLH), and *PGSC0003DMG400001339* (MYB), indicating dynamic reorganization of regulatory control depending on stress type. In contrast, the *S. lycopersicum* networks exhibited a more compact structure, with a single hub under heat stress (*Solyc11g011260*, GRAS) and three under drought (*Solyc06g036170*, GRAS; *Solyc10g086530*, GRAS; *Solyc10g052470*, MYB-related). Together with the overrepresented promoter motifs shared between the species, these findings point to a multi-layered transcriptional regulation involving diverse TF families, consistent with our previous results [46]. However, it is worth noting that our study predicted possible associations between genes and transcription factors, based on three complementary sources of evidence: coexpression, protein–protein interactions, and enrichment of regulatory motifs in their promoter regions. Therefore, the putative roles of these transcription factors in mediating drought and heat responses require experimental validation. Thus, while both crops activate overlapping sets of protective pathways, the architecture and hierarchy of transcriptional control appear to have diverged during evolution and domestication.

A closer look at antioxidant systems regulation shows both specialized and shared strategies that *S. lycopersicum* and *S. tuberosum* use when facing abiotic stresses. When tomato plants experience heat stress, they increase expression of certain electron transport components, particularly 2Fe-2S ferredoxin-type and chloroplastic ferredoxin-1 proteins, along with gamma-glutamylcyclotransferase. This suggests an adaptive approach to managing photosynthetic electron flow while maintaining glutathione homeostasis during thermal stress [80]. This compartmentalized response fits with the fundamental principle that plant antioxidant systems are highly organized across different cellular spaces, with chloroplasts serving as the main ROS production sites requiring specialized, locally-acting defense mechanisms [81]. Interestingly, many classical ROS-scavenging enzymes, including glutathione reductase, multiple thioredoxins, and COQ6, are actually downregulated during heat stress. This might indicate a shift from eliminating ROS toward using ROS as signaling molecules that help fine-tune stress responses [82]. This nuanced regulation appears evolutionarily conserved, as shown by orthologous genes behaving similarly across species. For example, chloroplastic ferredoxin I (*Solyc01g103920* and *PGSC0003DMG400004532*) is consistently upregulated under heat in both tomato and potato, highlighting its fundamental role in redox buffering during thermal challenges. In contrast, drought stress triggers a different antioxidant profile: both species strongly increase expression of specific peroxidases, *Solyc02g092580* in tomato and *PGSC0003DMG401001731* in potato, emphasizing peroxidase-mediated hydrogen peroxide clearance as a central drought tolerance mechanism [83]. The stress-specific nature of these responses becomes even clearer when looking at repressed genes, which show minimal overlap between heat and drought conditions. This suggests plants have distinct redox remodeling programs for different environmental challenges [84]. Notably, our analysis reveals three evolutionarily conserved, stress-responsive orthologs with indirect antioxidant functions, germin-like proteins and dihydrolipoyl dehydrogenase, that are consistently downregulated under drought across both species. This pattern could represent strategic metabolic trade-offs that prioritize resource allocation toward immediate fight against ROS and maintaining full defense capacity [85]. Therefore, heat stress seems to favor redox signaling and electron redistribution pathways, while drought emphasizes enzymatic ROS detoxification. This sophisticated stress-specific tuning likely underlies the different growth resilience and ROS management observed between species facing distinct environmental challenges.

In the context of our comparative genomic and transcriptomic analyses, we also observed marked evolutionary divergence among paralogous genes in *S. lycopersicum*, *S. pennellii*, and *S. tuberosum* (Figure 4C), contrasted by strong evolutionary conservation among orthologs (Figure 4D), particularly between *S. lycopersicum* and *S. pennellii*. The number of stress-induced orthogroups varied by a factor of 1.5–2 between these species (Figure 4A), while the overlap of orthologs within the most relevant DEG-based regulatory networks of heat and drought response between *S. lycopersicum* and *S. tuberosum* did not exceed 7–10% (Supplementary Table S7). Furthermore, pronounced differences were detected in the composition of regulatory motifs and transcription factor families contributing to these networks. Taken together, these results indicate that genomic divergence within Solanaceae and the diversification of their molecular regulatory systems both contribute substantially, and possibly to a comparable extent, to the evolution of stress response mechanisms at the transcriptomic level. Therefore, a comprehensive systems-level approach that integrates regulatory, functional, and evolutionary perspectives represents the most promising strategy for uncovering the molecular foundations of stress adaptation in this plant family.

Placing our findings in a broader evolutionary context, recent pan-genomic analyses of the *Solanum* genus have demonstrated that extensive gene duplication and subsequent paralogue diversification are key drivers of transcriptional and functional divergence among related crops [52]. Even between the closely related species *S. tuberosum* and *S. lycopersicum*, such paralogous expansions can obscure genotype-to-phenotype relationships, providing a likely explanation for the species-specific transcriptional architectures observed in our study. Complementary evidence from the wild tomato *S. chilense* further suggests that drought-responsive gene networks differ in evolutionary age and transcriptional stability [86]. Core regulatory modules associated with cell cycle control are evolutionarily ancient and highly conserved, while metabolic and signaling networks are of more recent origin and display greater variability. Together, these insights indicate that the evolution of abiotic stress responses in *Solanum* involves both structural diversification through gene duplication and functional divergence in the timing and dynamics of stress-related network evolution. This combination of conserved regulatory backbones and flexible peripheral modules likely underlies the balance between robustness and adaptability that characterizes abiotic stress responses in *S. tuberosum* and *S. lycopersicum*.

When viewed in the context of earlier transcriptomic studies, our results reinforce and extend existing models of stress-induced regulation in Solanaceae. In agreement with prior work, heat stress in *S. tuberosum* is characterized by the upregulation of genes related to photosynthesis, cell wall modification, RNA processing, and protein turnover [87], whereas drought stress induces genes involved in carbohydrate metabolism, flavonoid and lipid biosynthesis, and secondary metabolism [88]. For *S. lycopersicum*, functional annotations of downregulated DEGs under heat stress emphasize cellular and metabolic processes, while heat shock proteins display pronounced activation [89]. Similarly, during drought response, tomato exhibits coordinated transcriptomic and proteomic regulation involving genes associated with general stress responses, abiotic stimuli, and oxidative balance [90]. Altogether, these findings suggest that *S. tuberosum* and *S. lycopersicum* share a conserved regulatory backbone but utilize distinct network architectures to achieve stress resilience, reflecting both their evolutionary divergence and contrasting physiological strategies.

From a broader perspective, the integration of orthology inference, network reconstruction, and meta-analysis presented here provides a new comparative framework for understanding abiotic stress adaptation in the Solanaceae. By resolving stress-responsive transcriptional networks at the cross-species level, our study bridges molecular and evolutionary scales of regulation, linking conserved orthologous modules to lineage-specific regulatory innovations. This integrative approach highlights the potential of systems-level analyses to uncover evolutionary constraints and plasticity within complex stress response networks.

In summary, our study establishes a comparative and evolutionary framework for linking gene network organization to abiotic stress adaptation within the Solanaceae. By integrating large-scale transcriptomic data, orthology analysis, and systems-level reconstruction, we identify conserved molecular signatures and reveal how evolutionary diversification of regulatory networks underpins species-specific strategies of drought and heat tolerance. These results advance our understanding of the molecular evolution of stress responses in plants and provide a solid foundation for future research in functional genomics and adaptive breeding of Solanaceae crops.

## 4. Materials and Methods

### 4.1. Pipeline for Identification and Systematic Analysis of Key Differentially Expressed Genes in Solanum tuberosum and Solanum lycopersicum Under Drought and Heat Stress

**Figure 7:**
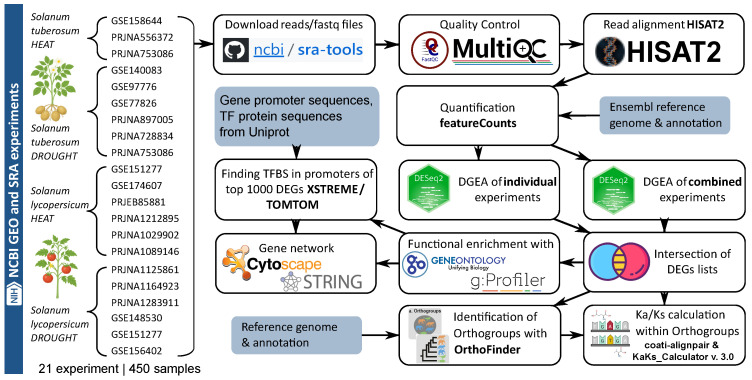
Overview of the collected datasets and the complete workflow used in the large-scale meta-analysis of drought and heat stress responses in *S. tuberosum* and *S. lycopersicum*. The schematic illustrates the main steps of the analysis, including data acquisition, quality control, read alignment, expression quantification, differential gene expression (DEG) analysis, functional enrichment of Gene Ontology (GO) terms, promoter motif analysis, and the construction of gene co-expression networks.

For the comprehensive large-scale analysis, the data were retrieved and processed through the following interconnected stages shown in the Figure 7.

### 4.2. Search and Selection of Relevant Transcriptomic Experiments for Analysis

Gene Expression Omnibus (GEO) was screened to find RNA-Seq datasets related to drought/water deficit and heat/high temperature stresses. To qualify for inclusion in the meta-analysis, an expression dataset had to meet the following criteria: (1) it’s raw RNA-seq reads are deposited in a public data repository; (2) it is collected under controlled conditions with replicates; (3) it is provided with metadata and method description. As a result, the total of 450 samples from 21 experiments for *S. tuberosum* and *S. lycopersicum* were selected for further analysis. All included studies, along with associated information, are listed in Table 1.

### 4.3. RNA-Seq Data Processing

The raw reads were downloaded in FastQC format from the NCBI SRA repository (https://www.ncbi.nlm.nih.gov/geo/, accessed on 10 October 2025) using prefetch and fasterq-dump from SRA Toolkit (https://github.com/ncbi/sra-tools, accessed on 10 October 2025, version 3.1.1, NCBI, Bethesda, MD, USA). Reads quality was checked using FastQC (https://www.bioinformatics.babraham.ac.uk/projects/fastqc/, accessed on 10 October 2025, version 0.11.9, Babraham Institute, Cambridge, Great Britain) and results were aggregated with MultiQC v1.27 [97]. FastQC reports indicated that the quality of the raw sequencing data submitted to the SRA was adequate for downstream analysis; therefore, no pre-processing or trimming of the raw expression data was required.

For *Solanum tuberosum* Solanum_tuberosum.SolTub_3.0.dna_sm.toplevel.fa reference genome sequences file and Solanum_tuberosum.SolTub_3.0.57.chr.gtf gene annotation files were obtained from the Ensembl Plants database in FASTA and GFF formats. For *Solanum lycopersicum* Solanum_lycopersicum.SL3.0.dna.toplevel.fa and Solanum_lycopersicum.SL3.0.57.chr.gff3 were retrieved from the Ensembl Plants database for reference genome and gene annotation files, respectively (https://ftp.ensemblgenomes.ebi.ac.uk/pub/plants/release-57/, accessed on 10 October 2025).

Reads were mapped to the reference genomes of *S. tuberosum* and *S. lycopersicum* using Hisat2 [98] (https://daehwankimlab.github.io/hisat2/, accessed on 10 October 2025, version 2.2.1, Johns Hopkins University, Baltimore, MD, USA) with –no-unal and –no-mixed flags, and alignments were converted to BAM with samtools v.1.9 [99]. Aligned reads were quantified using featureCounts [100] with the following parameters: assign reads to all their overlapping meta-features (-O), set the minimum mapping quality score to 10 (-Q), set attribute type to ‘Parent’ (-g).

### 4.4. Differential Gene Expression Analysis

Two complementary approaches were applied to identify differentially expressed genes (DEGs) associated with drought and heat stress responses. (1) In the first approach, DEGs were identified within each individual experiment, and the resulting DEG lists were combined and ranked according to the frequency with which a gene appeared as a DEG across multiple transcriptomic experiments. (2) In the second approach, samples from all relevant experiments were merged into four combined datasets (two stress types × two species), and batch effects associated with experiment identifiers (“GSE/Project” variable) were adjusted by including this factor in the design formula. DEGs were then identified from the combined datasets.

For the first approach, pairwise comparisons were performed separately within each experiment to avoid confounding by batch effects (e.g., comparisons were made independently for different cultivars or stress durations).

For the second approach, the following design formula was applied to the DESeq2 model: design = GSE + stress, where the “GSE” term accounts for variation between projects, and the “stress” term captures the biological effect of interest. Since DESeq2 [101] uses a generalized linear model (GLM) to fit read counts and iteratively estimate β coefficients for each experimental factor, the effect of the unwanted variable (“GSE”) is controlled by fitting its own β coefficient. The β coefficient corresponding to the stress factor thus represents the estimated $\log_{2}$ fold change (LFC).

In both approaches, DEGs were identified using the negative binomial model implemented in DESeq2 [101] on the raw gene count matrix generated by featureCounts. The contrasts were defined as “Heat vs Control” and “Drought vs Control.” A $\log_{2}$ fold change ≥0.32 threshold was applied during hypothesis testing. The independent filtering of low-count genes was left at its default setting. To mitigate inflation of LFC values for lowly expressed genes, LFC shrinkage was performed using the adaptive shrinkage estimator (type = “ashr”). Genes with an *s*-value (estimated false sign rate) below 0.005 and an absolute $\log_{2}$ fold change ≥0.32 were considered significantly differentially expressed.

For downstream analyses, the intersection of DEGs identified by both approaches was used. For each species–stress combination, the top 1000 DEGs (top 500 upregulated and top 500 downregulated genes) from this intersection were selected. Genes were then ranked by the absolute value of their LFC within each occurrence group. Each group was defined by the number of pairwise comparisons in which a gene was identified as a DEG across individual experiments. For example, in *S. tuberosum* under drought stress, the top 500 upregulated DEGs included genes that were consistently detected as differentially expressed in multiple independent comparisons (from 19 down to 13). Within each occurrence group, genes were then sorted by decreasing absolute LFC, so that the most strongly upregulated genes appeared first.

### 4.5. Enrichment Analysis and Functional Annotation

Functional enrichment analysis of lists of differentially expressed genes was done with the gprofiler2 R library (https://doi.org/10.32614/CRAN.package.gprofiler2, accessed on 10 October 2025). Genes without functional annotation (having the “gene of unknown function” mark in the annotation file) were searched in non-redundant NCBI protein database using blastp.

Functional enrichment analysis was performed using the clusterProfiler package in R (v. 4.4.1) to identify overrepresented Gene Ontology (GO) terms among the top differentially expressed genes (DEGs) in each species–stress combination.

### 4.6. Promoter Analysis and Motif Identification

Promoter analysis was performed to identify conserved cis-regulatory motifs potentially involved in the transcriptional regulation of drought- and heat-responsive genes in *S. tuberosum* and *S. lycopersicum*. Upstream promoter sequences were extracted from reference genome annotations using bedtools flank and bedtools getfasta [58], based on gene coordinates from Ensembl Plants release 57 (https://ftp.ensemblgenomes.ebi.ac.uk/pub/plants/release-57/, accessed on 10 October 2025).

Motif discovery was carried out using the XSTREME tool from the MEME Suite, and identified motifs were annotated with TOMTOM (https://meme-suite.org/). Only motifs with a *q*-value < 0.05 were retained for further analysis.

To associate the identified motifs with putative transcription factors (TFs), protein sequences of known TFs were retrieved from UniProt and aligned against the *S. tuberosum* and *S. lycopersicum* proteomes using blastp (parameters: max_target_seqs = 10, evalue = 0.01). BLAST v2.15.0+ results were filtered according to the following thresholds: minimum sequence identity of 50%, maximum difference in aligned sequence length of 30%, and minimum coverage of 50% for both query and target sequences. The resulting matches were used to infer potential regulatory associations between conserved promoter motifs and candidate stress-responsive transcription factors in both species.

### 4.7. Orthogroup Identification and Evolutionary Analysis

Comparative genomic analysis was performed to identify orthologous gene groups and to characterize evolutionary relationships among Solanaceae species, including wild potato accessions. Predicted proteomes from 11 plant genomes were analyzed, comprising eight reference Solanaceae genomes obtained from the PLAZA database (https://bioinformatics.psb.ugent.be/plaza.dev, accessed on 10 October 2025) and three wild potato genomes (*Solanum neorossii* PG6243, *S. pinnatisectum* PG1013, and *S. burkartii* PG4005 reported in [60]).

Protein-coding sequences were extracted using gffread v. 0.12.8 [102] from genome FASTA and GFF annotation files. Transcripts lacking valid start or stop codons, or containing internal stop codons, were removed, and overlapping isoforms were merged into single loci to obtain nonredundant proteome datasets.

Orthologous relationships were inferred using OrthoFinder v. 3.0.1b1 [59] with default parameters. Orthogroup clustering produced both standard and hierarchical orthogroup (HOG) assignments, and a species tree was inferred from concatenated multiple-sequence alignments of single-copy orthologs generated during the OrthoFinder run. Summary statistics, including total gene number, proportion of genes assigned to orthogroups, and number of species-specific orthogroups, were calculated automatically and verified using in-house R scripts (R v. 4.3.1) with the tidyverse, ggtree, and cowplot packages.

Selective constraints among orthologous genes were assessed using pairwise nonsyno nymous-to-synonymous substitution rate ratios ($K_{a}/K_{s}$) calculated with KaKs_Calculator v. 3.0 [103]. Coding sequences from each orthogroup were aligned with coati-alignpair (https://github.com/CartwrightLab/coati, accessed on 10 October 2025) using the Muse and Gaut codon model and the Gotoh alignment algorithm. Only hierarchical orthogroups containing genes from all six representative Solanaceae genomes (*Capsicum annuum*, *Nicotiana tabacum*, *Petunia axillaris*, *Solanum lycopersicum*, *S. pennellii*, and *S. tuberosum*) were retained for downstream analysis. Gene pairs with $K_{s}>0.01$ or coding sequence length shorter than 300 bp were excluded to minimize artifacts due to saturation and low-quality alignments.

The resulting $K_{a}/K_{s}$ distributions were used to evaluate patterns of purifying and neutral selection across orthogroups. All data processing, statistical analyses, and visualization were performed in the same R environment. Details on analyzed genomes, orthogroup composition, and sequence data sources are provided in Supplementary Files S3 and S4.

### 4.8. Gene Network Reconstruction, Analysis, and Visualization

GRNs were reconstructed for the top 500 upregulated and top 500 downregulated differentially expressed genes (DEGs) identified in *Solanum tuberosum* under drought stress and in *Solanum lycopersicum* under heat stress. For *S. lycopersicum* under drought conditions, a total of 440 DEGs were identified, including 369 downregulated genes, all of which were included in the network reconstruction. The smaller number of DEGs in this case reflects the limited sample size and the lower statistical power of available transcriptomic datasets for drought-treated *S. lycopersicum*, which constrained the detection of significant expression changes.

Three sources of evidence were integrated to build the networks:

1. **Motif-based regulatory associations.** Statistically enriched promoter motifs specifically associated with subsets of up- and downregulated genes were identified using MEME/XSTREME (see Section 4.6). These motifs were used to infer potential transcriptional regulatory links.
2. **Coexpression relationships.** Gene coexpression networks were reconstructed using the WGCNA package [104]. Adaptive correlation thresholds were applied depending on the number of experiments within each subset [47]:
  - *S. lycopersicum* (drought): threshold 0.4, 6 experiments;
  - *S. lycopersicum* (heat): threshold 0.4, 6 experiments;
  - *S. tuberosum* (drought): threshold 0.4, 6 experiments;
  - *S. tuberosum* (heat): threshold 0.9, 3 experiments.
  WGCNA was performed with the following settings: an unsigned network (networkType = “unsigned”), soft-thresholding power chosen by the pickSoftThreshold function to attain scale-free topology, unsigned TOM (TOMType = “unsigned”), average linkage hierarchical clustering for gene dendrogram construction, dynamic module detection (cutreeDynamic) with deepSplit = 2 and a minimum module size of 30 genes, and module merging based on eigengene correlation > 0.75.
3. **Protein–protein interactions (PPIs).** Experimentally validated and database-curated interactions were retrieved from STRING (https://string-db.org/, accessed on 10 October 2025) [105]. Protein identifiers were imported using the proteins by sequences option, with the following parameters: evidence sources were set to experiments and databases, and the minimum interaction score was defined as medium confidence (0.400).

All three interaction layers were merged into integrated GRNs and visualized in Cytoscape v. 3.10.3 (https://cytoscape.org/, accessed on 10 October 2025) [106]. Initial layouts were generated using the Edge-weighted Spring Embedded algorithm, followed by Circular Layout optimization to enhance cluster visualization. Clusters were defined based on connection types and prioritized as follows: (1) coexpression edges, (2) motif-based regulatory associations, and (3) PPIs for genes lacking direct regulatory links. Clusters were numbered in descending order according to the number of genes they contained. Functional enrichment analysis for each cluster was performed using Gene Ontology Biological Process terms in ShinyGO v. 0.85 (https://bioinformatics.sdstate.edu/go/, accessed on 10 October 2025) [107]. The complete list of enriched Gene Ontology terms identified across all four experimental conditions is provided in Supplementary File S6.

To identify orthologous genes within the reconstructed regulatory networks, we used the Ensembl BioMart Plants service (https://plants.ensembl.org/info/data/biomart/index.html, accessed on 10 October 2025) [108] and the *Ensembl Plant Genes 62* dataset.

## 5. Conclusions

In this study, we integrated transcriptomic and comparative genomic analyses to identify key genes and regulatory mechanisms underlying drought and heat stress responses in *Solanum tuberosum* and *Solanum lycopersicum*. By applying two complementary differential expression strategies, individual experiment analysis and combined dataset meta-analysis, we generated a robust and reproducible set of stress-responsive genes while minimizing experiment-specific variability. The integration of these approaches revealed both species-specific and conserved transcriptional responses: tomato exhibited a more focused heat-responsive expression profile, whereas potato displayed broader transcriptional activation under drought conditions. The functional annotation of differentially expressed genes demonstrated the activation of cellular protection mechanisms, including chaperone-mediated protein folding, oxidative stress mitigation, and crosstalk with immune signaling pathways. Downregulated genes were enriched in metabolic and photosynthetic processes, indicating energy reallocation toward stress tolerance and survival. Analysis of promoter motifs and transcription factor (TF) classes highlighted the central roles of bZIP, bHLH, DOF, and BBR/BPC families in regulating heat- and drought-responsive transcriptional programs, underscoring the multilayered organization of regulatory networks in Solanaceae. Comparative genomic analysis established the evolutionary framework for these transcriptional patterns. Potato genomes exhibited lineage-specific gene duplications and a higher proportion of species-specific orthogroups compared to tomato and other Solanaceae species, suggesting that recent duplication events contributed to the diversification of stress adaptation pathways. Together, these results indicate that both evolutionary diversification and transcriptional plasticity contribute to the stress resilience of Solanaceae species. Overall, our findings provide an integrated view of the molecular and evolutionary mechanisms underlying drought and heat stress responses in two major Solanaceae crops. This work establishes a reference framework for identifying candidate genes, regulatory elements, and conserved adaptive modules that can inform future functional studies and crop improvement strategies targeting environmental stress resilience. Our proposed framework for analyzing gene and gene network evolution enables large-scale prediction of key genes important for breeding and hybridization. We note the potential for further in-depth study of stress-induced orthogroups and the accumulation of substitutions within them, which will complement the overall picture of evolutionary trends. The obtained results reveal complex patterns in evolution and open new avenues for further research.

## Figures and Tables

**Table 1:** Included studies, along with associated information.

GEO/Project ID	Number of Samples ^1^	Reference
*Solanum tuberosum*		
heat stress:		
GSE158644	12	[39]
PRJNA556372	18	[38]
PRJNA753086	18	—
drought stress:		
GSE140083	24	[33]
GSE97776	54	[34]
GSE77826	48	[35]
PRJNA897005	20	[36]
PRJNA728834	21	[37]
PRJNA753086	17	—
*Solanum lycopersicum*		
heat stress:		
GSE151277	15	[41]
GSE174607	12	[91]
PRJEB85881	98	[92]
PRJNA1212895	6	—
PRJNA1029902	6	—
PRJNA1089146	6	[93]
drought stress:		
GSE148530	8	[42,43]
GSE151277	18	[41]
GSE156402	12	[94]
PRJNA1125861	27	[95]
PRJNA1164923	4	[96]
PRJNA1283911	6	—

^1^ Indicates the number of samples included in the analysis (i.e., some samples were omitted due to low coverage or other issues).

## Data Availability

The original contributions presented in this study are included in the article/Supplementary Material. Further inquiries can be directed to the corresponding author(s).

## Supplementary Materials

The following supporting information can be downloaded at: https://www.mdpi.com/article/10.3390/plants14243851/s1: Supplementary File S1: Summary of the RNA-seq experiments used in this study. The metadata include accession identifiers, number of samples, sequencing layout and platform, plant cultivar, tissue type, growth conditions, duration of stress treatment, and links to the corresponding article/source of the data. Supplementary File S2: Differential gene expression analysis (DEA) results performed on Solanum tuberosum and Solanum lycopersicum datasets under drought and heat stress conditions. The file includes 12 worksheets organized as follows: 4 sheets present results of DEA for pairwise comparisons within individual experiments; 4 sheets contain results of DEA for combined datasets representing meta-analysis across experiments; and 4 sheets summarize the intersections of differentially expressed genes identified by both individual and combined analyses. Supplementary File S3: Genomes used for orthology inference. This table lists the genomes used as input for orthology inference with OrthoFinder, and provides links to the sources of reference proteoms. Supplementary File S4: Orthology inference results. This file corresponds to the OrthoFinder N0.tsv output, summarizing orthogroup and hierarchical orthogroup assignments across 11 analyzed species. Each row represents an orthogroup, and columns correspond to species included in the analysis. Entries list gene identifiers belonging to each orthogroup. Supplementary File S5: Nonsynonymous/synonymous substitution rate ($K_{a}/K_{s}$) substitution rate analysis. This file reports the $K_{a}/K_{s}$ calculated using KaKs_Calculator v3.0 for a subset of six selected genomes. $K_{a}$ and $K_{s}$ values were computed for all pairwise alignments within orthogroups (gene composition of orthogroups can be found in Supplementary File S4). Supplementary File S6: Lists of Gene Ontology enriched terms in *S. tuberosum* and *S. lycopersicum* clusters of GRNs in response to heat and drought. Supplementary File S7: Lists of presented orthologous in reconstructed GRN in response to drought and heat stress in *S. tuberosum* and *S. lycopersicum*.

## Abbreviations

The following abbreviations are used in this manuscript:

GEO	Gene Expression Omnibus
ROS	reactive oxygen species
DEGs	differentially expressed genes
GRN	gene regulatory network
ABA	abscisic acid
LFC	$\log_{2}$ fold change
TF	transcription factor
GO	Gene Ontology
HOGs	hierarchical orthologous groups
HSF	heat shock transcription factors
